# Targeting FCRLA to induce necrosis in lung adenocarcinoma: a novel strategy for prognosis and therapy via MPT-Driven pathways

**DOI:** 10.3389/fimmu.2025.1596179

**Published:** 2025-08-13

**Authors:** Xiaoli Sun, Bowen Cai, Shusen Zhang, Xiaowei Cao, Zhen Wang, Zhigang Cai

**Affiliations:** ^1^ The First Department of Pulmonary and Critical Care Medicine, The Second Hospital of Hebei Medical University, Shijiazhuang, Hebei, China; ^2^ Department of Pulmonary and Critical Care Medicine, Shijiazhuang People's Hospital, Shijiazhuang, Hebei, China; ^3^ Hebei Key Laboratory of Respiratory Critical Care Medicine, Shijiazhuang, Hebei, China; ^4^ Hebei Institute of Respiratory Diseases, Shijiazhuang, Hebei, China; ^5^ Department of Pharmacy, The Second Hospital of Hebei Medical University, Shijiazhuang, Hebei, China; ^6^ Department of Pulmonary and Critical Care Medicine, Xing tai People’s Hospital, Xingtai, Hebei, China

**Keywords:** lung adenocarcinoma, mitochondrial permeability transition driven necrosis, FCRLA, risk model, new strategy

## Abstract

**Introduction:**

The induction of mitochondrial permeability transition-driven necrosis (MPTDN) is therapeutically relevant in various cancers. However, few studies have explored the role of MPTDN-related genes (MPTDNRGs) in lung adenocarcinoma (LUAD). Therefore, this study investigated the regulatory mechanisms of MPTDNRGs in LUAD.

**Methods:**

This study was based on The Cancer Genome Atlas-Lung Adenocarcinoma (TCGA-LUAD), GSE31210, and MPTDNRGs. First, the genes obtained from TCGA-LUAD were intersected through differential expression analysis and weighted gene co-expression network analysis (WGCNA) to obtain the candidate *FCRLA* gene. An *FCRLA* knockdown cell model was constructed *in vitro* using LUAD cells, and cell-related phenotypic experiments, including proliferation and apoptosis, were performed. The integrity of the mitochondrial structure was observed using electron microscopy, and the mitochondrial membrane potential was detected using a JC-1 probe.

**Results:**

A total of 82 candidate genes were identified by intersecting 3,231 differentially expressed genes with 566 key module genes. Subsequently, three prognostic genes (*RASGRP2*, *CD79A*, and *FCRLA*) were further screened. *CD79A* and *FCRLA* were significantly expressed in the LUAD group, whereas the opposite was true for *RASGRP2*. *In vitro* studies indicated that *FCRLA* knockdown significantly inhibited the proliferation of LUAD cells and induced necrosis in these cells. Electron microscopy found that the mitochondrial structure was disrupted after *FCRLA* knockdown. The JC-1 probe indicated that the mitochondrial membrane potential in the *FCRLA*-knockdown group was significantly reduced, suggesting impaired mitochondrial function.

**Discussion:**

*RASGRP2*, *CD79A*, and *FCRLA* have been identified as being associated with MPTDN in LUAD cells. *FCRLA* knockdown may suppress mitochondrial permeability transition through specific pathways, thereby driving LUAD cell necrosis and providing potential targets for subsequent LUAD treatment.

## 1 Introduction

Lung cancer is a primary global health problem and remains the most common cancer type and the foremost cause of cancer-related deaths in China (1). The Agency for Research on Cancer reported approximately 820,000 new lung cancer diagnoses and 715,000 lung cancer-related deaths in China in 2020 (1, 2). Non-small cell lung cancers (NSCLCs) are classified based on histopathological characteristics. The most common histological subtype of NSCLC is adenocarcinoma, which accounts for approximately 55–60% of cases in Chinese patients (3). Notably, the 5-year overall survival for all patients with all types of lung cancer is only approximately 19%. In China, the incidence and mortality of lung cancer differ substantially between different sexes, ages, and regions (4), with great variability and heterogeneity among the different subtypes, ranging from 24% for patients with NSCLC to 6% in small cell lung cancer. Given this heterogeneity and the high incidence of lung adenocarcinoma (LUAD), identifying prognostic markers for LUAD is essential.

The mitochondria play a crucial role in regulating the homeostasis of cancer cells and programmed cell death (5). When mitochondria are damaged, stress or stimulation occurs, and the permeability of the mitochondrial inner membrane increases rapidly, leading to mitochondrial dysfunction and ultimately, cell apoptosis (6). Mitochondrial permeability transition (MPT) plays a role in mitochondria-mediated apoptosis (7). Several MPT regulatory mechanisms are impaired in tumors compared to surrounding tissues. MPT inhibition may be a mechanism that protects the survival and proliferation of cancer cells (8). A study by West China Hospital confirmed that targeting the opening of MPT pores enhances nanoparticle drug delivery and mitigates cancer metastasis (9). Furthermore, adenovirus-mediated mda-7 overexpression (10) resulted in the rapid induction of apoptosis in both p53-resistant and p53-sensitive lung cancer cells. This mechanism appears to involve the release of MPT pores, which subsequently induces cell death. Thus, necrosis driven by MPT may be an attractive strategy for developing novel cancer therapies.

This study identified prognostic genes associated with MPT-driven cell necrosis in LUAD, based on transcriptome data, and constructed related prognostic models using *FCRLA*, *CD79A*, and *RASGRP2*. It verified whether the expression differed in the prognostic model in tissues and LUAD cell lines. Bioinformatics methods were used to explore the relationship between it and immune infiltration, as well as the impact of immunotherapy. Based on these results, *FCRLA*, which showed the greatest correlation with the immune checkpoints, was selected for further analysis. Genes of the Fc receptor - like (FCRL) family play a crucial role in the pathogenesis and progression of cancer. FCRL is regarded as a potential target for cancer treatment (11). These findings supported our hypotheses. The effects of *FCRLA* knockdown on tumor cell proliferation, apoptosis, mitochondrial morphology, and mitochondrial membrane potential were studied. Thus, a new prognostic model was established for patients with LUAD, providing a theoretical basis for exploring the role of MPT-driven necrotic genes in the development of LUAD. These results are also of great significance for the prognosis and treatment of patients, providing novel insights and directions for the treatment of other cancers.

## 2 Materials and methods

### 2.1 Data collection

The training set was The Cancer Genome Atlas-Lung Adenocarcinoma (TCGA-LUAD) dataset from the University of California Santa Cruz Xena (https://xenabrowser.net/datapages/), which was used to analyze 01A and 11A, containing 510 LUAD tissues and 58 control paraneoplastic tissue samples, and 497 samples from the LUAD samples with survival information were used to construct the risk model. The GSE31210 and GSE13213 datasets were downloaded as a validation set from the Gene Expression Omnibus database (https://www.ncbi.nlm.nih.gov/geo/). The GSE31210 dataset contained 246 LUAD samples with survival information from 226 cases, whereas the GSE13213 dataset contained 117 LUAD samples. In addition, 39 MPT-driven necrosis-related genes (MPTDNRGs) were derived from the M17902, M3873, and M16257 gene sets in the Gene Set Enrichment Analysis-Molecular Signatures Database (https://www.gsea-msigdb.org/gsea/msigdb).

### 2.2 Differential expression analysis

Differentially expressed genes (DEGs) between LUAD and control samples were analyzed using the R package DESeq2 (version 1.40.2) (12) in TCGA-LUAD with a filter of |log2fold change (FC)| > 1 and P.adjust < 0.05. Afterwards, volcano plots and heat maps were visualized using the R packages ggplot2 (version 3.4.2) (13) and circlize (version 0.4.15) (14), respectively.

### 2.3 Weighted gene co-expression network analysis

Module genes associated with MPTDNRGs ARGs were identified using weighted gene co-expression network analysis (WGCNA). First, the scores of MPTDNRGs in all samples were calculated using the single-sample gene set enrichment analysis algorithm of the R package GSVA (version 1.48.3) (15). The Wilcoxon test was then performed to compare the LUAD and control groups and determine whether a significant difference existed. The samples were then clustered using the R package WGCNA (version 1.72-1) (16) to identify any outliers that needed to be excluded. The soft threshold (β) was determined when the mean connectivity tends to 0 after eliminating outlier samples; this ensures that the genes interact in a way that best fits the scale-free distribution. The minimum number of genes per gene module was set to 200 in accordance with the standards of the dynamic tree-cutting algorithm. Finally, the MPTDNRGs score was used as the phenotypic trait, and each module was subjected to Pearson’s correlation analysis (|Cor| > 0.3 and P-value < 0.05). The selected key module had the highest correlation, and the key module genes were used for subsequent analyses. The LUAD samples were divided into high- and low-score groups based on the MPTDNRGs scores, and the Kaplan–Meier (KM) survival curve was drawn between the groups using the R package Survminer (version 0.4.9) (17).

### 2.4 Enrichment analysis and construction of protein–protein interaction network

The R package Eulerr (version 7.0.0) (18) was used to intersect DEGs and key module genes to identify candidate genes. To identify common functions and related pathways, the R package clusterProfiler (version 4.8.2) (19) (P.adjust < 0.05) was used to perform Gene Ontology (GO) and Kyoto Encyclopedia of Genes and Genomes (KEGG) enrichment analyses of candidate genes, and the R package Treemap (version 2.4-4) (20) was used to visualize the enrichment results. In addition, a protein–protein interaction (PPI) network was constructed for the candidate genes using the STRING database (https://cn.string-db.org/) (confidence score> 0.4) to explore potential interactions between the candidate genes.

### 2.5 Screening for prognostic genes

In the TCGA-LUAD, the candidate genes were subjected to univariate Cox analysis to screen the candidate prognostic genes with HR ≠ 1 and P < 0.05. Then, the candidate prognostic genes satisfying the PH hypothesis test (P > 0.05) were entered into the least absolute shrinkage and selection operator regression analysis to identify the optimal log (Lambda) values and their corresponding genes, which were defined as the prognostic genes, and the Wilcoxon signed rank test was used to verify the expression of prognostic genes in TCGA-LUAD.

### 2.6 Building and validating the risk model

Prognostic gene expression and overall survival information were used to construct a risk model for TCGA-LUAD. To further assess the effectiveness of the risk model, a calibration curve was constructed to evaluate its predictive accuracy. Receiver operating characteristic (ROC) curves for 1, 3, and 5 years were plotted using the survival ROC of the R package (version 1.0.3.1) (21). The following formula was used to obtain the risk score for prognostic genes:


$$R\text{isk score}=\sum_{\text{n}=1}^{\text{n}}\left(\text{coefi}*\text{Xi}\right)$$

The samples were then divided into high- and low-risk groups, based on the median risk score. The KM curves of the two risk groups were drawn using the Survminer R package (version 0.4.9) (17), and the log-rank test (P < 0.05) was used to compare survival differences between the two groups. Furthermore, to evaluate the universality of the risk model, the GSE31210 and GSE13213 datasets were used to verify its accuracy. The risk model divides high and low risk groups using the same cutoff value sur_cut$cutpoint, and the cutoff value was calculated using the function “survminer” (0.5.0) (https://CRAN.R-project.org/package=survminer) to obtain the best cutoff point.

### 2.7 Independent prognostic analysis

The risk score and clinical features (node, stage, sex, age, tumor) were analyzed using univariate Cox analysis to obtain independent candidate prognostic factors (HR ≠ 1 and P < 0.05). After meeting the PH hypothesis test (P > 0.05), a multivariate Cox analysis was performed to identify independent prognostic factors (HR ≠ 1 and P < 0.05). Subsequently, the R package rms (version 6.7-0) (22) was used to construct a nomogram model based on independent prognostic factors, and the predictive ability of the nomogram model was assessed using ROC, calibration, and decision curve analysis (DCA). The GSE13213 dataset was used for external verification. To further investigate the relationship between clinical features and risk score, the difference in risk score was calculated, and a Kruskal–Wallis test was performed among the five clinical feature subgroups mentioned above (P < 0.05).

### 2.8 Analysis of enrichment of risk groups

For a deeper understanding of the potential biological mechanisms and pathways involved between the two risk groups, the R package DESeq2 was used to perform differential analysis between the two risk groups. Based on the log2FC value as a sorting criterion, Gene Set Enrichment Analysis (GSEA) enrichment analysis based on the KEGG pathway background gene set “c2.cp.kegg.v2023.1. Hs.symbols” in MSigDB (https://www.gsea-msigdb.org/gsea/msigdb) was performed using the R package clusterProfiler (P < 0.05). The variability in the enriched pathways between the two risk groups was compared using Gene Set Variation Analysis (GSVA). First, on the basis of the background gene set “h.all.v2023.1. Hs.symbols.gmt” in MSigDB (https://www.gsea-msigdb.org/gsea/msigdb), the R package GSVA (version 1.48.3) (15) was used to calculate the GSVA score for each sample in the risk groups. The individual enriched entries were then analyzed for differences between the two risk groups using the limma package (version 3.56.2) (23), with P.adjust < 0.05 as the threshold.

### 2.9 Immunological correlation analysis

The tumor microenvironment is valuable for the diagnostic and prognostic assessment of tumors. First, the relationship between the risk score and six immune infiltrating cell types (macrophages, CD8 T cells, neutrophils, B cells, CD4 T cells, and dendritic cells) was assessed based on the Tumor Immune Estimation Resource database (https://cistrome.shinyapps.io/timer/) (|cor| > 0.3 and P < 0.05). Second, variability in 16 immune cells, 13 immune-related functional aspects (24), 11 immune checkpoints (25), and three scores (StromalScore, ImmuneScore, and ESTIMATEScore) were compared between the two risk groups based on the TCGA-LUAD dataset. Three scores, immune-related function scores, and immune cell types of LUAD samples were calculated using the single-sample gene set enrichment analysis algorithm of the R package GSVA (version 1.48.3) (15) and the R package estimates (version 1.0.13) (26), respectively. Ultimately, correlations between prognostic genes and differential immune checkpoints, as well as the relationship between the three scores and the risk score, were explored using Spearman’s correlation analysis (|cor| > 0.3, P.adjust < 0.05).

### 2.10 Construction of the *FCRLA* knockdown cell model and experimental grouping

Human LUAD cell lines A549 and NCI-H1975 were purchased from Procell Life Science & Technology Co., Ltd. (Wuhan, China). RPMI 1640 medium (G4532, Servicebio, China) was supplemented with 10% fetal bovine serum (G8003, Servicebio) and 1% penicillin–streptomycin (G4003, Servicebio) and prewarmed at 37°C. The cells were thawed in liquid nitrogen and placed in a 37°C water bath (Shanghai Jingqi Instrument Co., Ltd., China) for 3 min, followed by the addition of fresh RPMI 1640 medium. The cells were then incubated in a cell culture incubator (Herocell 180, RADOBIO, China) at 37°C with 5% CO_2_ overnight. The culture medium was refreshed every two days. Cells in the logarithmic growth phase were used for subsequent experiments.

A549 and NCI-H1975 cells in the logarithmic growth phase were selected to construct *FCRLA*-knockdown LUAD cell lines. Specific siRNAs targeting FCRLA were designed and synthesized. Lipid-based transfection reagents were used to transfect siRNAs into A549 and NCI-H1975 cells, according to the manufacturer’s instructions. After 72 h of transfection, the expression levels of *FCRLA* were detected using RT-qPCR and western blotting to verify knockdown efficiency. The siRNA sequences are listed in **Supplementary Table S1**. In the model validation experiment for *FCRLA* knockdown, three groups were used: control, si-NC, and si-FCRLA. Three groups, namely si-NC, si-FCRLA, and si-FCRLA + 1 μmol/L cyclosporin A (HY-B0579, MedChemExpress, China), were used in the experiment to verify the association between knocking down *FCRLA* and the MPT pathway. Cyclosporin A effectively inhibited cyclophilin D (CypD) protein expression, and its concentration was determined as previously described (27).

### 2.11 CCK8 assay

A549 and NCI-H1975 cells in the exponential growth phase were obtained and incubated with trypsin solution (G4013, Servicebio) for 3 min, followed by centrifugation at 1200 × *g* for 3 min. The trypsin solution was discarded, and fresh medium was added to resuspend the cells. Cell density was calculated using a cell counter (G8003, Servicebio). The cells were seeded at a density of 3 × 10^3^ cells/well in 96-well plates and incubated overnight. After treating the cells according to the requirements of the different groups, the cells were incubated for an additional 48 h. Then, 10 μL of CCK8 solution (CA1210, Servicebio, China) was added to each well, and the cells were placed in a 37°C, 5% CO2 cell incubator for 3 h. The 96-well plates were then removed, and the OD value at 450 nm was measured using a microplate reader (ReadMax 1900Plus, Flash, China).

### 2.12 EDU staining

The cells were transfected, digested, centrifuged, and cell counts were performed as previously described. Cells were seeded in six-well plates at a density of 1 × 10^5^ cells/well and incubated overnight. The next day, EDU working solution (E-CK-A377, Elabscience Biotechnology Co., Ltd, China) was added to the cell culture medium, and the cells were incubated for an additional 2 h. After incubation, the medium containing EDU was aspirated, and the cells were washed with phosphate-buffered saline (PBS). Next, the cell fixative was added and incubated for 30 min. DAPI (C1005, Beyotime, China) was added, and the cells were incubated in the dark for an additional 5 min. They were then washed with PBS. After washing with PBS, the cells were observed and photographed using a confocal microscope (STELLARIS, Leica, Germany).

### 2.13 Hoechst 33342/PI staining

Cells were processed as previously described, seeded in six-well plates at a density of 1 × 10^5^ cells/well, and incubated overnight. The next day, the cell culture medium was aspirated, and the cells were washed with PBS. Next, the cell fixative (P1110, Servicebio) was added, and the cells were incubated for 30 min. Then, Hoechst 33342 (BL116A, Biosharp, China) staining solution was added and incubated in the dark for 10 min, followed by the addition of PI staining solution and incubation in the dark for an additional 5 min. The cells were washed with PBS, and after washing, they were observed and photographed for analysis under a confocal microscope.

### 2.14 Flow cytometry to detect cell apoptosis

Cells were processed as previously described, seeded in six-well plates at a density of 2 × 10^5^ cells/well, and incubated overnight. The next day, the cell culture medium was aspirated, the cell pellet was obtained as described previously, and the cells were centrifuged after digestion. PBS was added to resuspend the cells, and the cells were incubated in the cell fixative for 5 min. According to the operation instructions for the apoptosis kit (C1062M, Beyotime), the cell fixative was discarded, Binding Buffer was added to resuspend the cells, 5 μL of FITC Annexin V reagent was added, and the cells were incubated at room temperature in the dark for 15 minutes. Then, 10 μL of PI staining solution was added, and the cells were incubated in the dark for 5 min. After incubation, flow cytometry was used for detection, and apoptosis was analyzed.

### 2.15 JC-1 mitochondrial membrane potential fluorescent probe

A suitable number of cells was cultured in 12-well plates with coverslips. After the cells were cultured overnight and allowed to return to their normal state, drug treatment was performed. The culture supernatant was aspirated per the manufacturer’s instructions (C2006, Beyotime). The cell fixative (P1110, Servicebio) was added for incubation for 20 min, 0.5% Triton X-100 (P0096, Beyotime) was added for permeabilization for 20 min, and the cells were sealed with a mounting solution containing anti-fluorescence quencher (P0131, Beyotime). The results were observed and photographed for analysis under a laser confocal microscope.

### 2.16 Transmission electron microscopy

Each group of cells was treated as previously described. They were then fixed with 2.5% glutaraldehyde for 2 h, followed by fixation with 1% osmium tetroxide for 1 h. Subsequently, they were dehydrated using a series of ethanol gradients. The samples were then placed in a mixture of embedding agent and propylene oxide (1:1) for permeation for 2 h, and subsequently placed in pure embedding agent for permeation for an additional 5 h. The samples were placed in an embedding mold for sectioning (60 nm). Uranium acetate and lead citrate staining were performed, and the morphological changes in mitochondria in the cells were observed using transmission electron microscopy (JEM-1400FLASH, Tokyo, Japan).

### 2.17 RT-qPCR experiment

Total RNA was extracted from cells in each group according to the manufacturer’s instructions for the Trizol kit (R0011, Beyotime). cDNA was synthesized using the cDNA synthesis kit (D7170L, Beyotime) at 37°C for 15 min and 85°C for 5 s. Specific primers for each factor were designed, and the reaction system was prepared according to the manufacturer’s instructions for BeyoFast™ SYBR Green qPCR Mix. The reaction program of the fluorescence quantitative PCR instrument (4351405, Thermo Fisher Scientific, Waltham, MA, USA) was set as follows: 95°C for 2 min, followed by 40 cycles of 95°C for 15 s and 60°C for 15 s. After the reaction, the CT value data were retained for subsequent statistical analysis. The relative expression of mRNA was calculated using the ΔΔCt method (28). The primer sequences are detailed in **Supplementary Table S1**.

### 2.18 Western blot experiment

After treating the cells according to different grouping requirements, cell pellets were obtained, and RIPA lysis buffer (P0013E, Beyotime) was added. The mixture was incubated on ice for 30 min. The protein concentration in each group was determined using a BCA protein quantification kit (P0009, Beyotime). The protein solution was transferred to a water bath (E0530; Beyotime) at 99°C and incubated for 15 min. An equal amount of 30 μg of protein from each group was added to the electrophoresis tank, and electrophoresis was performed at a constant voltage of 120 V for 2 h. Proteins in the gel were transferred to a polyvinylidene fluoride membrane at a constant current of 260 mA for 1 h. The membrane was incubated with skim milk powder (P0216; Beyotime) for 2 h and incubated overnight with the corresponding primary antibody solution. The following day, the sections were incubated with the secondary antibody solution for 2 h, washed three times with TBST solution (ST673, Beyotime) for 30 min each, incubated with a chemiluminescent hypersensitive solution for 10 s, and then placed in a chemiluminescence imaging exposure system to obtain images. The gray values of the bands were statistically analyzed using ImageJ software (version 1.5.2a), and the ratio of the target gene to β-actin was used as an indicator of the relative expression of the target gene protein. FCRLA (1:1000, E-AB-53172, Elabscience Biotechnology Co., Ltd), β-actin (1:1000, ab8226, Abcam, China), CypD (1:1000, ab231155, Abcam); goat anti-rabbit IgG H&L (1:10000, ab205718, Abcam), and goat anti-mouse IgG H&L (1:10000, ab205719, Abcam) antibodies were used.

### 2.19 Statistical analysis

Data analysis of online databases was performed using the R software (https://www.r-project.org/) for statistical analysis. Differences between groups were analyzed using the Wilcoxon test or the Kruskal–Wallis test, with P-adjusted values < 0.05 representing a significant difference. *In vitro* cell experiments were analyzed using GraphPad Prism 9.5.0. One-way analysis of variance was used to evaluate all *in vitro* cell experiments, followed by *post-hoc* comparison using Tukey’s *post-hoc* significant difference test. All experiments were repeated at least three times.

## 3 Results

### 3.1 Acquisition of DEGs and key modular genes

**Figure 1:**
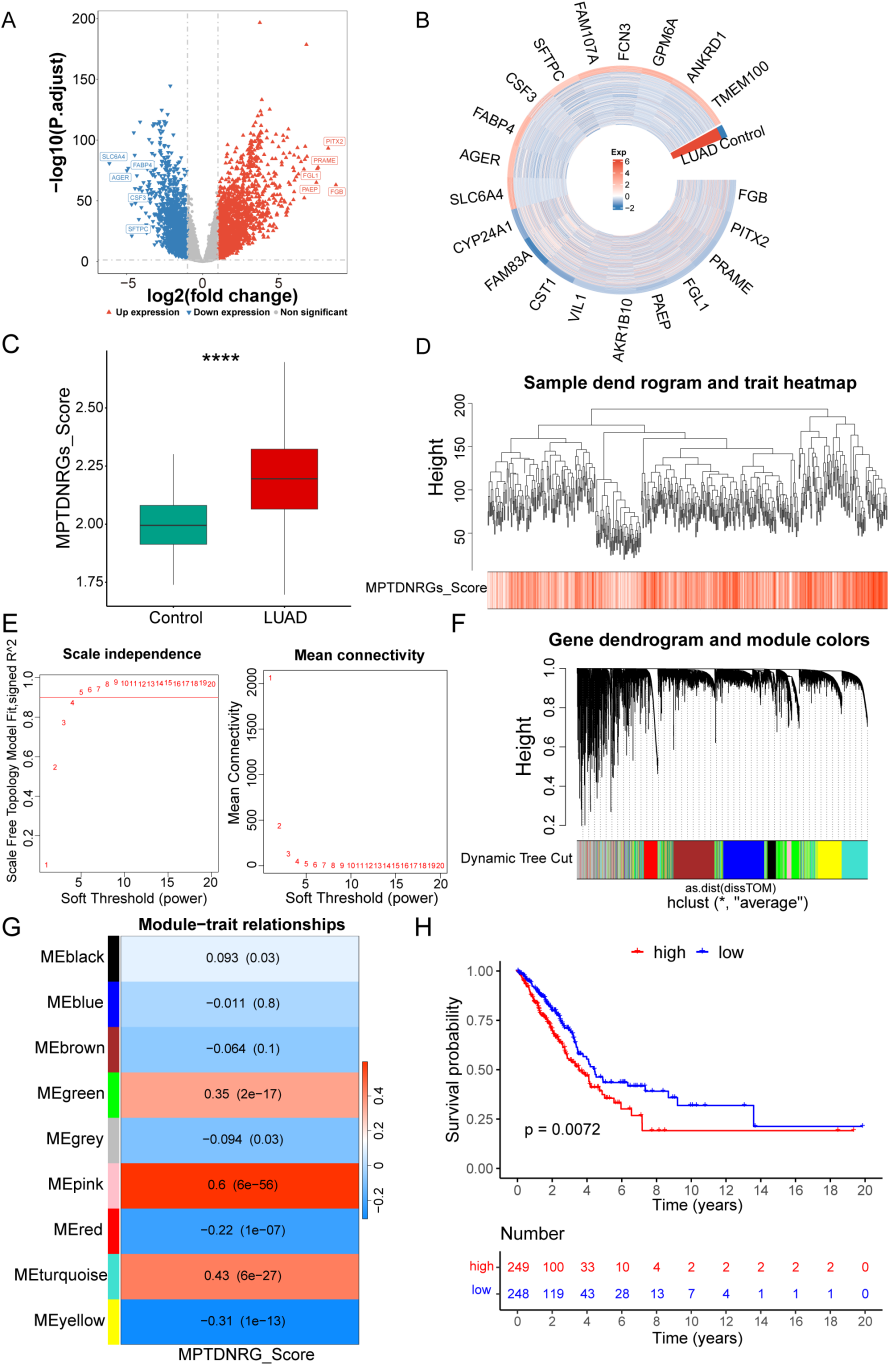
Differential gene analysis. **(A)** Volcano plot of differential genes in the differentially expressed genes (DEGs) dataset. **(B)** Heatmap analysis of DEGs. **(C)** Intergroup differences in the mitochondrial permeability transition-driven necrosis-related gene (MPTDNRG) scores. **(D)** Using the weighted gene co-expression network analysis (WGCNA) package in R, samples were clustered based on gene expression information in the training set, and the clustering situation was visualized. **(E)** Optimal soft threshold screening. **(F)** Dynamic shearing tree module. **(G)** Heatmap of the correlations between modules and traits. **(H)** Patient samples were divided into high- and low-score groups based on the MPTDNRGs score, and a Kaplan–Meier (KM) survival curve was generated between the groups using the Survminer package in R. *P < 0.05, ****P < 0.0001.

A total of 3,231 DEGs were identified between the LUAD and control groups, including 1,818 upregulated genes and 1,413 downregulated genes (**Figures 1A, B**). MPTDNRGs scores indicated that the LUAD group had a significantly higher score than the control group (**Figure 1C**). WGCNA was performed using the MPTDNRGs score as a trait to identify genes associated with MPTDNRGs. Clustering of the TCGA-LUAD samples revealed no outlier samples (**Figure 1D**). When R2 = 0.9, as well as β = 5, the scale-free distribution of the interaction between genes was maximized (**Figure 1E**). Based on dynamic tree cutting, nine modules were obtained (**Figure 1F**). The pink module, which contained 566 key module genes, showed the highest correlation with MPTDNRGs scores (Cor = 0.6, P < 0.001) (**Figure 1G**). Based on the MPTDNRGs scores, LUAD samples were divided into high- and low-expression groups, and the KM curve showed that the survival status of the two expression groups differed significantly (**Figure 1H**).

### 3.2 Identifying and analyzing candidate genes

**Figure 2:**
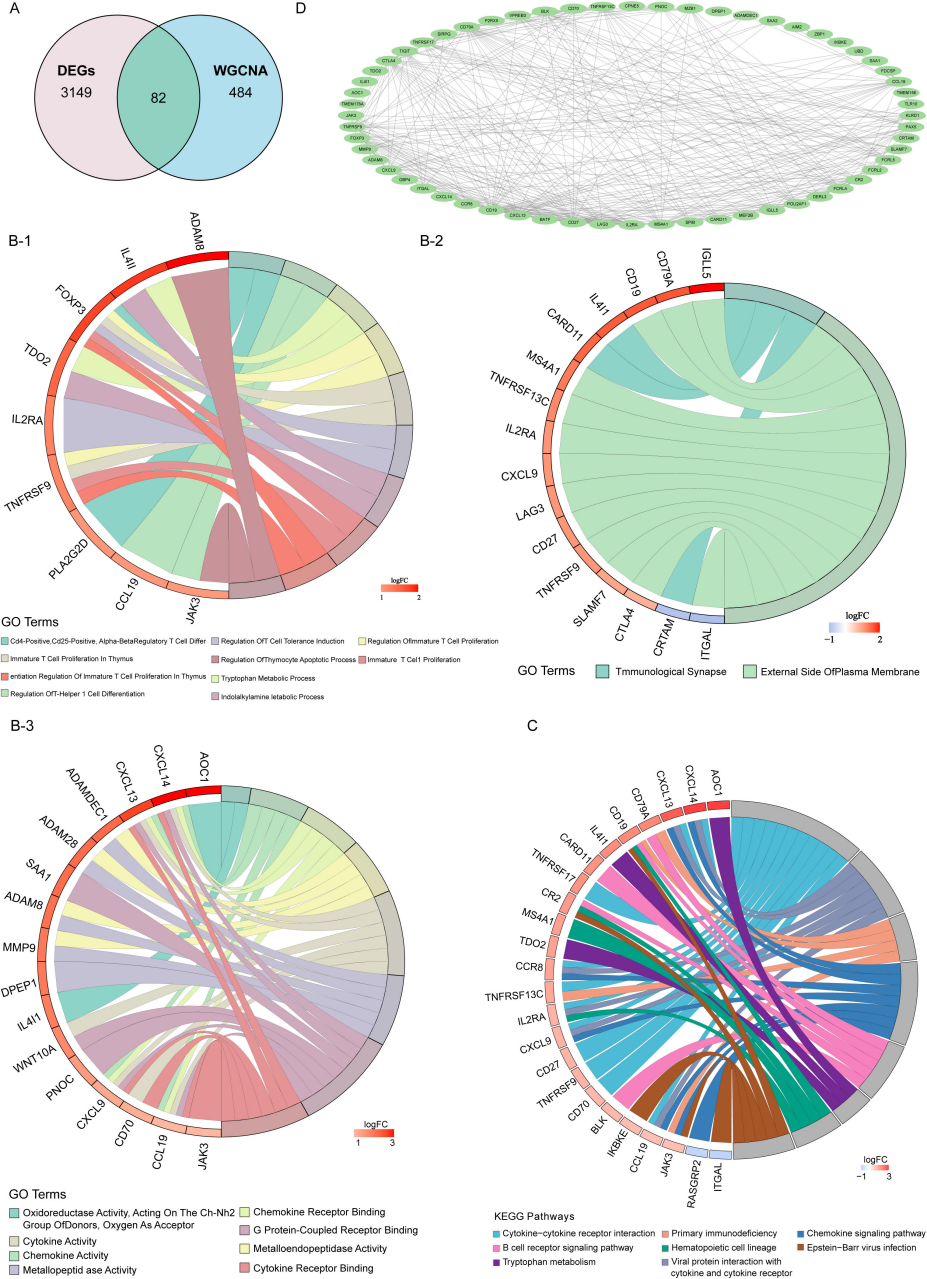
Identification of candidate genes. **(A)** The intersection of DEGs and module genes from WGCNA was obtained using the Eulerr package in the R language. **(B)** Gene Ontology (GO) enrichment analysis chord diagram of the candidate genes. **(B–1)** BP, **(B–2)** CC, **(B–3)** MF. **(C)** Chord diagram of the Kyoto Encyclopedia of Genes and Genomes (KEGG) enrichment analysis of candidate genes. **(D)** Protein–protein interaction (PPI) network of candidate genes.

A total of 82 candidate genes were identified by intersecting the DEGs with key module genes (**Figure 2A**). Candidate genes were enriched to identify related pathways and common functions, revealing a total of 294 GO entries and eight KEGG pathways. In GO, candidate genes were primarily enriched in mononuclear cell differentiation, the external side of the plasma membrane, chemokine activity, and other functional categories (**Figure 2B**) (**Supplementary Table S2**). In KEGG, AOC1 was significantly upregulated in tryptophan metabolism, whereas ITGAL and RASGRP2 were significantly downregulated in Epstein–Barr virus infection and chemokine signaling pathways, respectively (**Figure 2C**) (**Supplementary Table S3**). In addition, a PPI network was constructed between the candidate genes to clarify their interactions at the protein level (**Figure 2D**). The network comprised 335 interactions between 60 candidate genes, with CD19 exhibiting the highest number of interaction pairs, including CD19-CD79A, CD19-BLK, and other pairs.

### 3.3 Significant differences between the LUAD and control groups in prognostic gene expression

**Figure 3:**
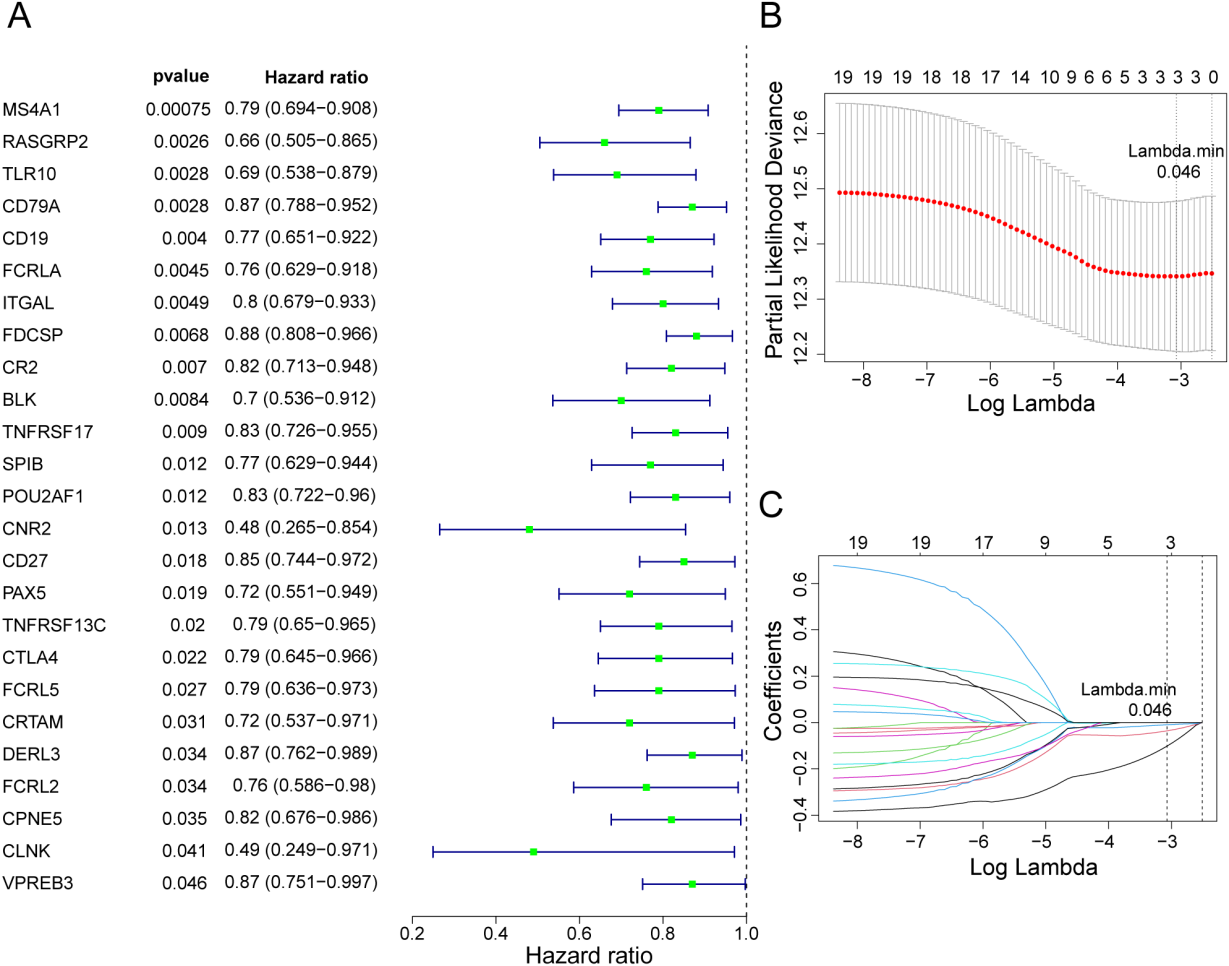
Candidate genes were selected based on DEG and MPTDNRG feature modules. **(A)** Univariate Cox regression analysis was performed for the 82 candidate genes. With HR ≠ 1 and a P < 0.05 as the criteria, 25 genes were screened, and the results are shown in a forest plot. **(B)** Least absolute shrinkage and selection operator regression analysis was performed on 19 genes screened using the univariate Cox + PH assumption test. **(C)** Expression of three prognostic genes in lung adenocarcinoma (LUAD) and control samples.

Univariate Cox regression analysis identified 25 candidate prognostic genes (**Figure 3A**), of which 19 met the PH hypothesis test. Least absolute shrinkage and selection operator regression analysis was used to further investigate three prognostic genes: *RASGRP2*, *CD79A*, and *FCRLA* (**Figures 3B, C**).

### 3.4 Better efficacy of the risk model

**Figure 4:**
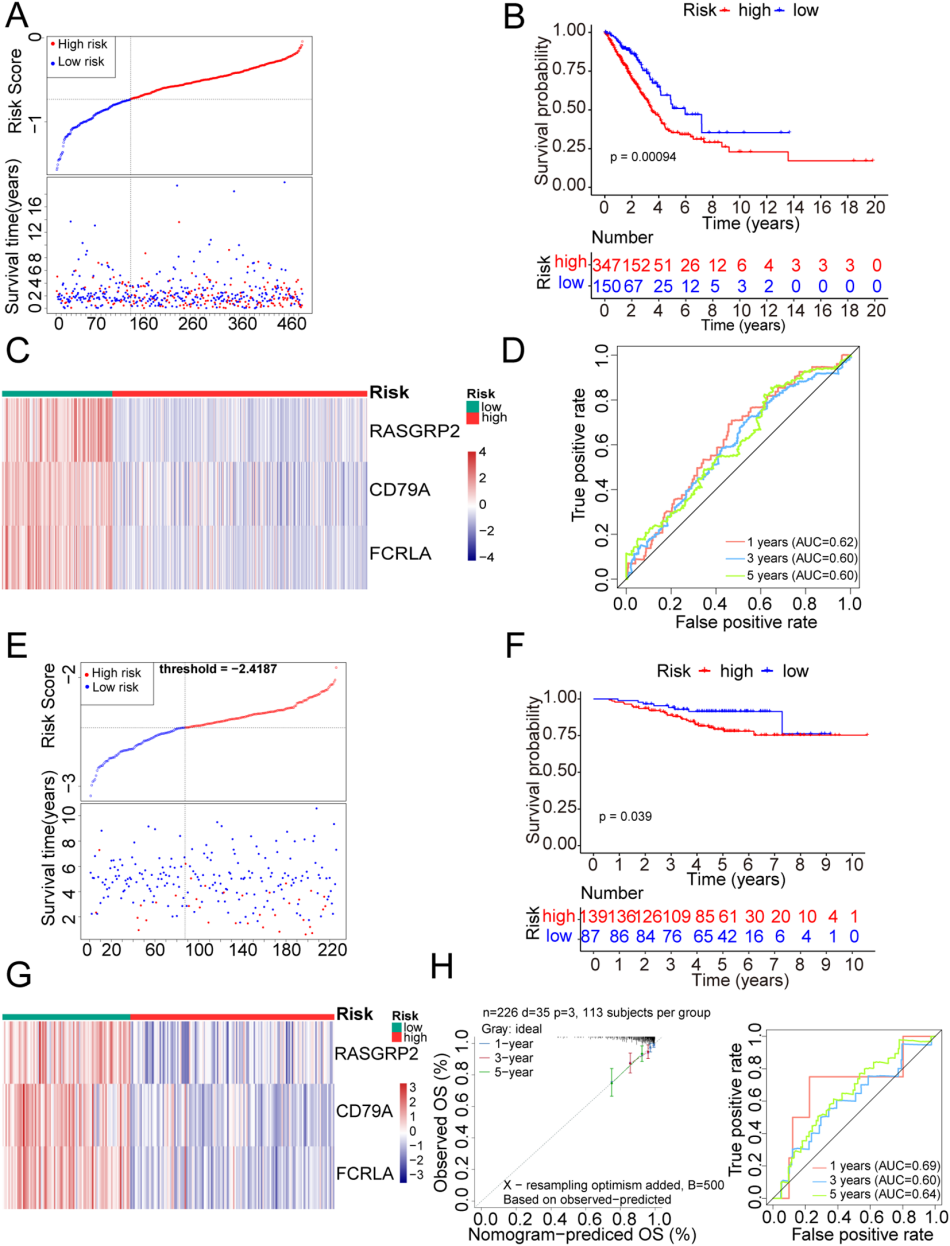
Construction of a prognostic risk model. **(A)** Using the median value of the risk score as the boundary, the samples were divided into two groups: high- and low-risk. After ordering the prognostic risk scores from low to high, a risk curve was constructed (TCGA dataset). **(B)** KM curve of the risk model (TCGA dataset). **(C)** Heatmap of prognostic gene expression in high- and low-risk groups (TCGA dataset). **(D)** The survivalROC package of R language was used to draw the receiver operating characteristic (ROC) curve. Based on the obtained prognostic risk score model, 1, 3, and 5 years were considered time nodes. **(E)** Based on the validation set, risk scores were sorted from low to high, and a risk curve was plotted. **(F)** Using the median value of the risk score as the boundary, the samples were divided into two groups: high- and low-risk. Differences in survival between the high- and low-risk groups were compared using a log-rank test. **(G)** Heatmap of prognostic gene expression in high- and low-risk groups. **(H)** ROC and survival curve drawing of the GSE31210 dataset of the Gene Expression Omnibus database. The calibration curve, with the predicted probability on the x-axis and the actual probability on the y-axis, indicates that the closer the slope of the calibration curve is to 1, the better the predictive performance of the model.

Based on the expression levels of the three abovementioned prognostic genes, a risk-scoring model was constructed for patients with LUAD. After dividing the patients into high- and low-risk groups based on the median risk value, the samples were divided into high- and low-risk groups. Both the risk (**Figure 4A**) and KM curves (**Figure 4B**) showed that individuals in the low-risk group had a lower mortality rate than those in the high-risk group; in the high-risk group, the prognostic genes were poorly expressed (**Figure 4C**). The AUC value of the ROC curve reached 0.6 at 1, 3, and 5 years (AUC1 = 0.62, AUC3 = 0.60, and AUC5 = 0.60) (**Figure 4D**), indicating that the risk model efficiency was enhanced. In other words, prognostic genes may be effective in predicting the survival status of patients with LUAD. Notably, the generalizability of the risk model was further validated using GSE31210, yielding consistent results with TCGA-LUAD (**Figures 4E–G**, **Supplementary Figures S1A–C**). GSE31210 ROC curve AUC values were 0.69, 0.60, and 0.64 at years 1, 3, and 5, respectively, and the slope of the calibration curve was close to 1 (**Figure 4H**). GSE13213 ROC curve AUC values were 0.71, 0.69, and 0.69 at years 1, 3, and 5, respectively (**Supplementary Figure S1D**).

### 3.5 Enhanced predictive power of the nomogram model

**Figure 5:**
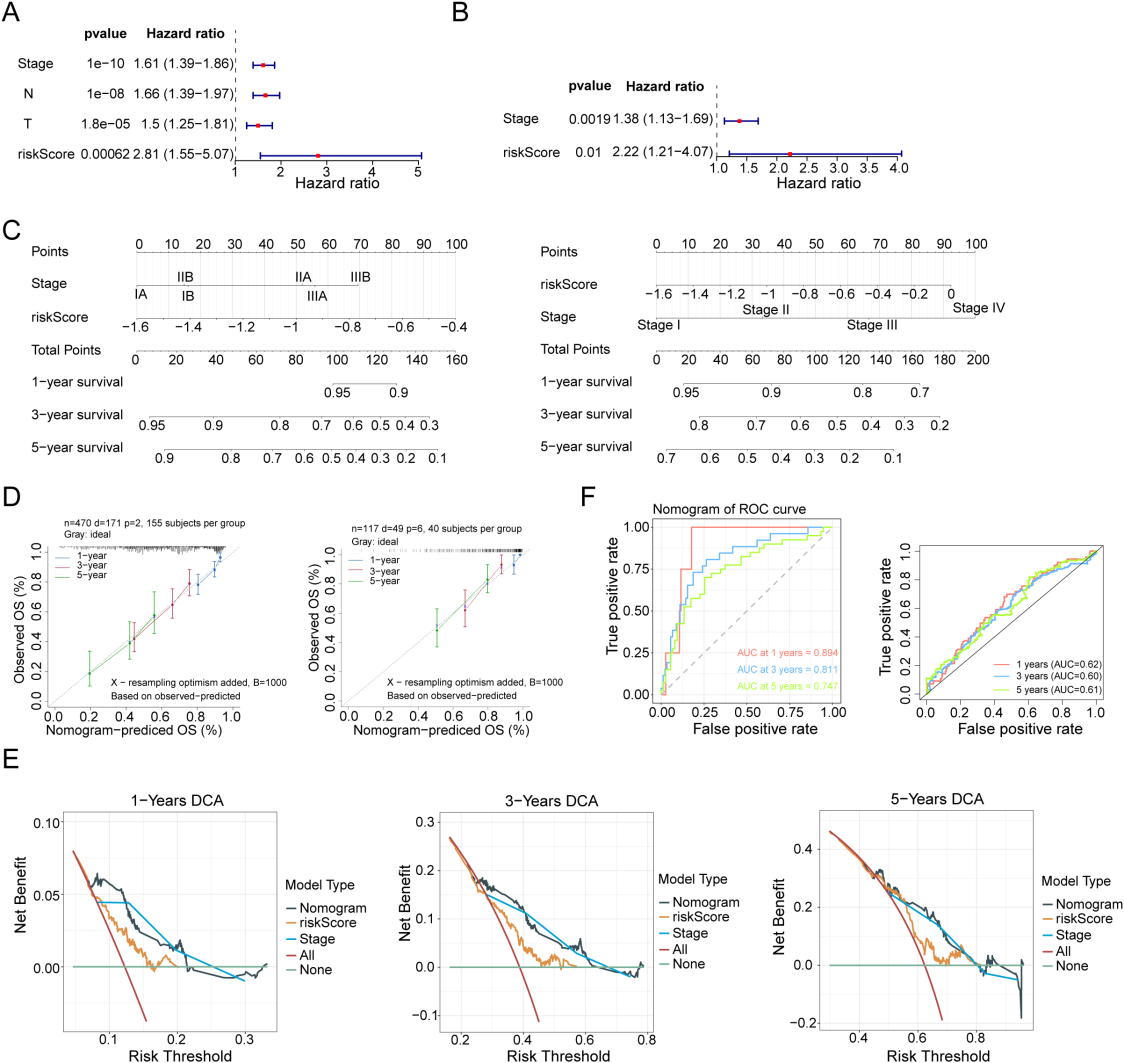
Construction of an independent prognostic model. **(A)** The six clinical characteristics of age, sex, stage, T, N, and risk score (risk model score) were simultaneously included in the univariate Cox analysis. Screening was performed with a P-value of < 0.05. **(B)** The stage and risk score obtained from the screening of univariate Cox + PH assumption tests were included in the multivariate Cox prognostic analysis. Screening was performed with a P-value of < 0.05. **(C)** Using the rms package of the R language, scoring was performed based on Stage and RiskScore. Each factor corresponds to a score, and the sum of the scores of each factor corresponds to the total score (total points). Subsequently, the 1-, 3-, and 5-year mortality rates were predicted based on the total score. The higher the score, the higher the mortality rate. Finally, the prediction results were drawn using a nomogram. **(D)** Calibration curve of the nomogram. **(E)** Decision curve analysis (DCA) curve of the nomogram model. **(F)** ROC curve.

**Figure 6:**
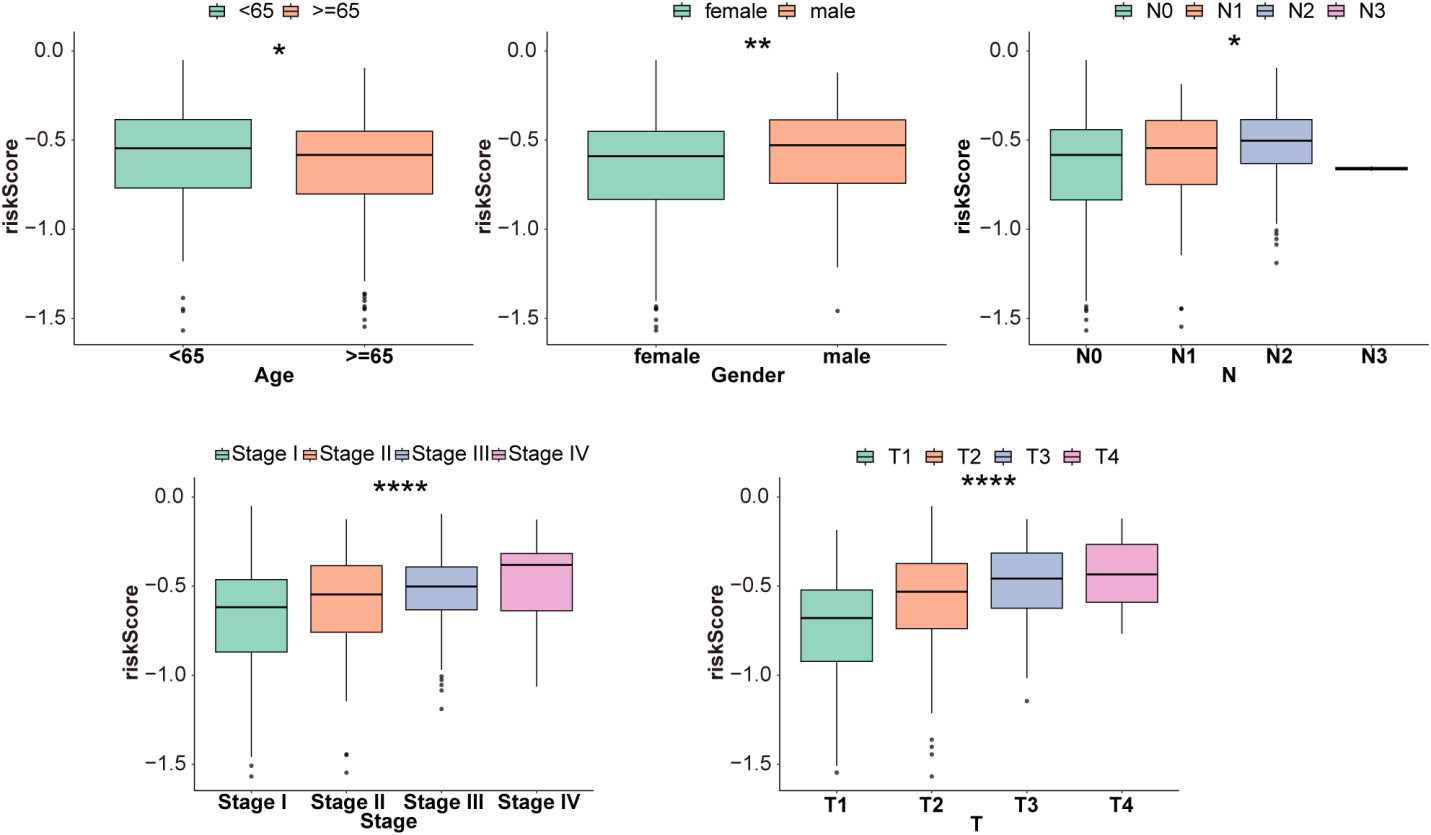
Risk score and clinical characteristics. A correlation analysis was performed between five clinical characteristics (age, sex, stage, T, and N) and the risk score. Differences in risk scores were calculated among the different clinical characteristic subgroups and subjected to the Kruskal–Wallis test. *P < 0.05, **P < 0.01, ****P < 0.0001.

Multivariate Cox regression analysis identified clinical stage (stage) and risk score (risk score) as independent prognostic factors (**Figures 5A, B**). Based on this, a nomogram model integrating these factors was constructed (**Figure 5C**) for individualized prediction of the survival rate of patients with LUAD. The slope of the calibration curve was close to 1 (**Figure 5D**), indicating a high degree of consistency between the predicted and actual survival rates. Decision curve analysis also showed that the model had a clinical net benefit at 1, 3, and 5 years (**Figure 5E**). The AUC value of the ROC curve reached 0.6 at 1, 3, and 5 years (GSE31210: AUC1 = 0.62, AUC3 = 0.60, and AUC5 = 0.61; GSE13213: AUC1 = 0.894, AUC2 = 0.811, and AUC3 = 0.747) (**Figure 5F**). These results suggest that the nomogram model exhibits better predictive ability. Moreover, the relationship between clinical features (N, stage, sex, T, and age) and risk score was further investigated, and analysis of variance showed that the risk score differed significantly between subgroups for each clinical feature (**Figure 6**).

### 3.6 Differential enrichment pathways between risk groups

**Figure 7:**
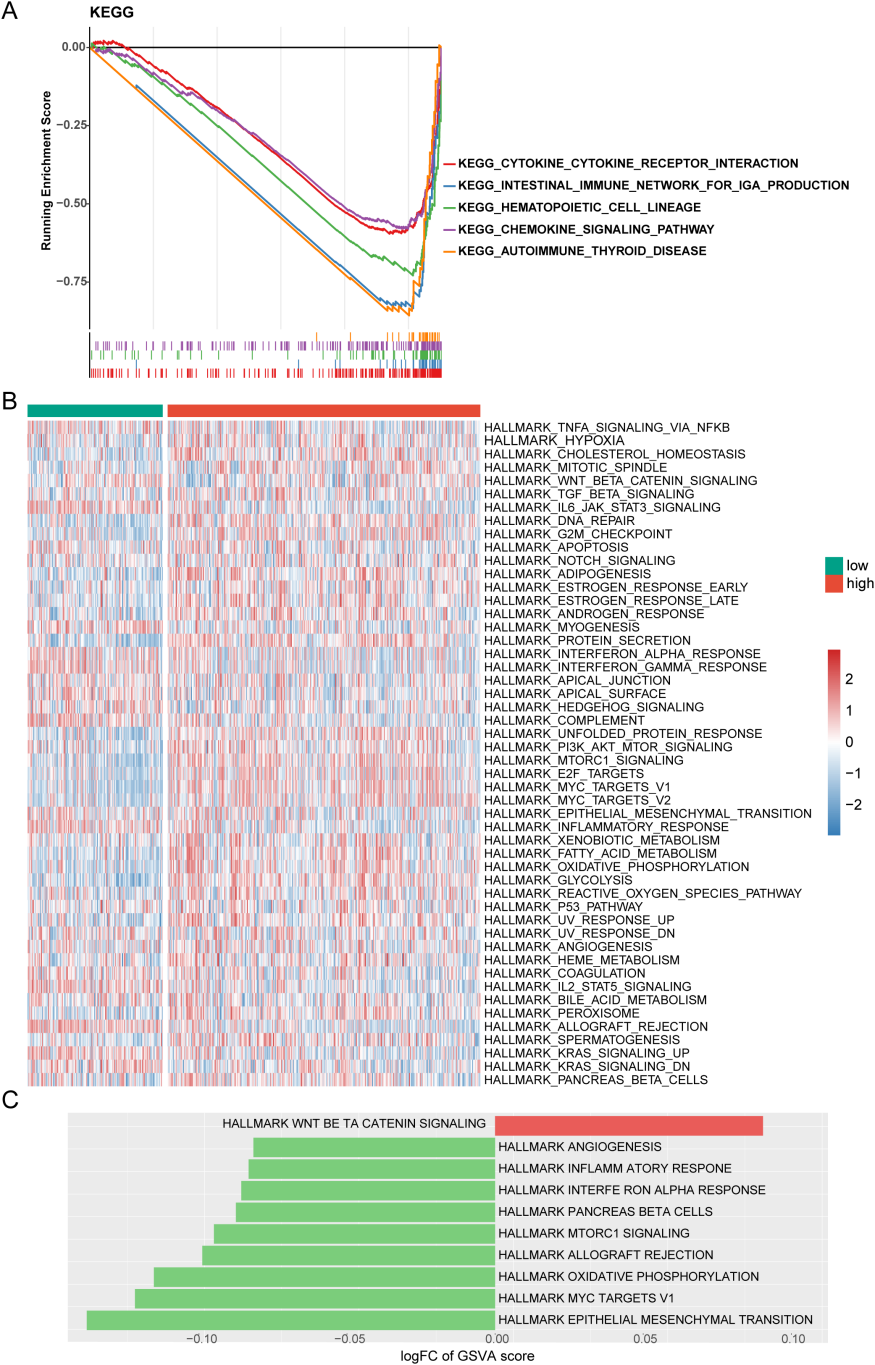
Enrichment analysis. **(A)** Gene Set Enrichment Analysis (GSEA) enrichment analysis in high- and low-risk groups **(B)** Gene Set Variation Analysis (GSVA) heatmap between high- and low-risk groups. **(C)** GSVA enrichment analysis between high- and low-risk groups.

To analyze the biological basis of risk grouping, GSEA and GSVA were performed to investigate the relevant signaling pathways and potential biological mechanisms underlying the differences between the two risk groups. GSEA showed that in the low-risk group, the hematopoietic cell lineage pathways, the intestinal immune network for IgA production, and cytokine–cytokine receptor interaction pathways were significantly enriched. In contrast, ribosomes were primarily enriched in the high-risk group (**Figure 7A**, **Supplementary Table S4**). The GSVA enrichment results and differential analysis between the two risk groups revealed nine downregulated pathways, including angiogenesis and inflammatory response, whereas only the Wnt beta-catenin signaling pathway was upregulated (**Figures 7B, C**, **Supplementary Table S5**).

### 3.7 Two risk groups differed in their response to immunization

**Figure 8:**
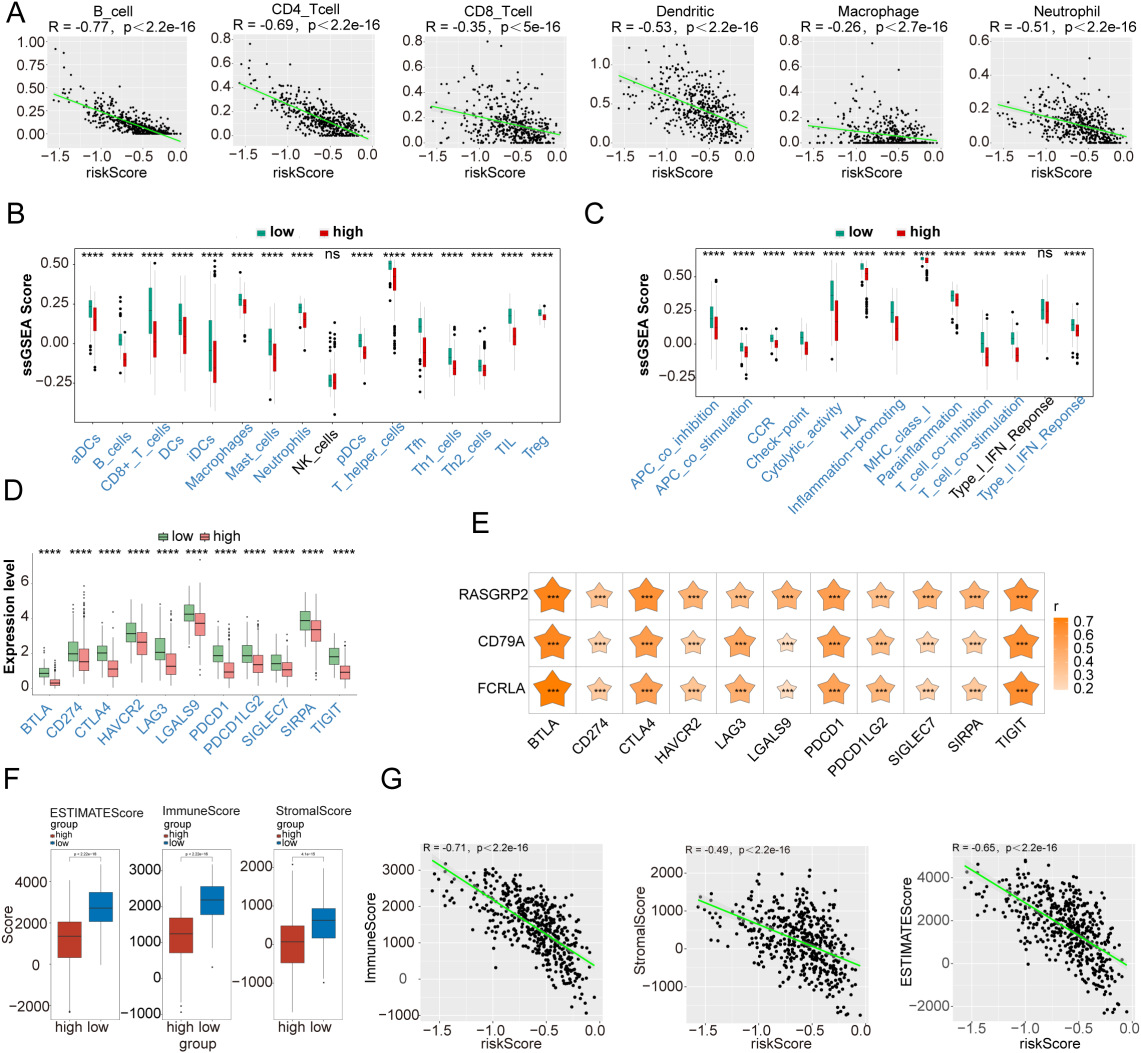
Immune infiltration analysis. **(A)** Correlation between the risk score and the infiltration levels of six immune cells (B cells, CD4 T cells, CD8 T cells, neutrophils, macrophages, and dendritic cells), and scatter plots. **(B)** Box plots of 16 immune cell types in the high- and low-risk groups. **(C)** Box plots of 13 immune-related functions in high- and low-risk groups. **(D)** Expression of immune checkpoint genes in high- and low-risk groups. **(E)** Heatmap of the correlation between the prognostic genes and immune checkpoints. **(F)** Box plots of tumor microenvironment scores. **(G)** Scatter plot of the correlation between the risk score and tumor microenvironment score. ***P < 0.001, ****P < 0.0001.

The correlations between the prognostic genes and the infiltration levels of various immune cells were investigated to evaluate the characteristics of the tumor immune microenvironment reflected by prognostic genes. The analysis revealed a significant correlation between the risk score and the infiltration levels of the five types of immune cells (excluding macrophages) (|cor| > 0.3, P < 0.05) (**Figure 8A**). Notably, an analysis of immune-related differences between the two groups at risk revealed that, except for natural killer cells and immune-related functions (type I interferon response), the remaining immune-related functions, immune cells, and immune checkpoints were expressed at higher levels in the low-risk group than in the high-risk group (**Figures 8B–D**). Spearman’s correlation analysis revealed the highest correlation between the prognostic gene *FCRLA* and the immune checkpoint BTLA (cor = 0.7358 and P < 0.001) (**Figure 8E**). Additionally, StromalScore, ImmuneScore, and ESTIMATEScore were also higher in the low-risk group than in the high-risk group (**Figure 8F**). A clear correlation existed between the risk score and all three scores (|cor| > 0.3 and P < 0.05), with the highest correlation observed with the Immune Score (cor = -0.71) (**Figure 8G**).

### 3.8 *FCRLA* knockdown inhibits the proliferation of lung cancer cells

**Figure 9:**
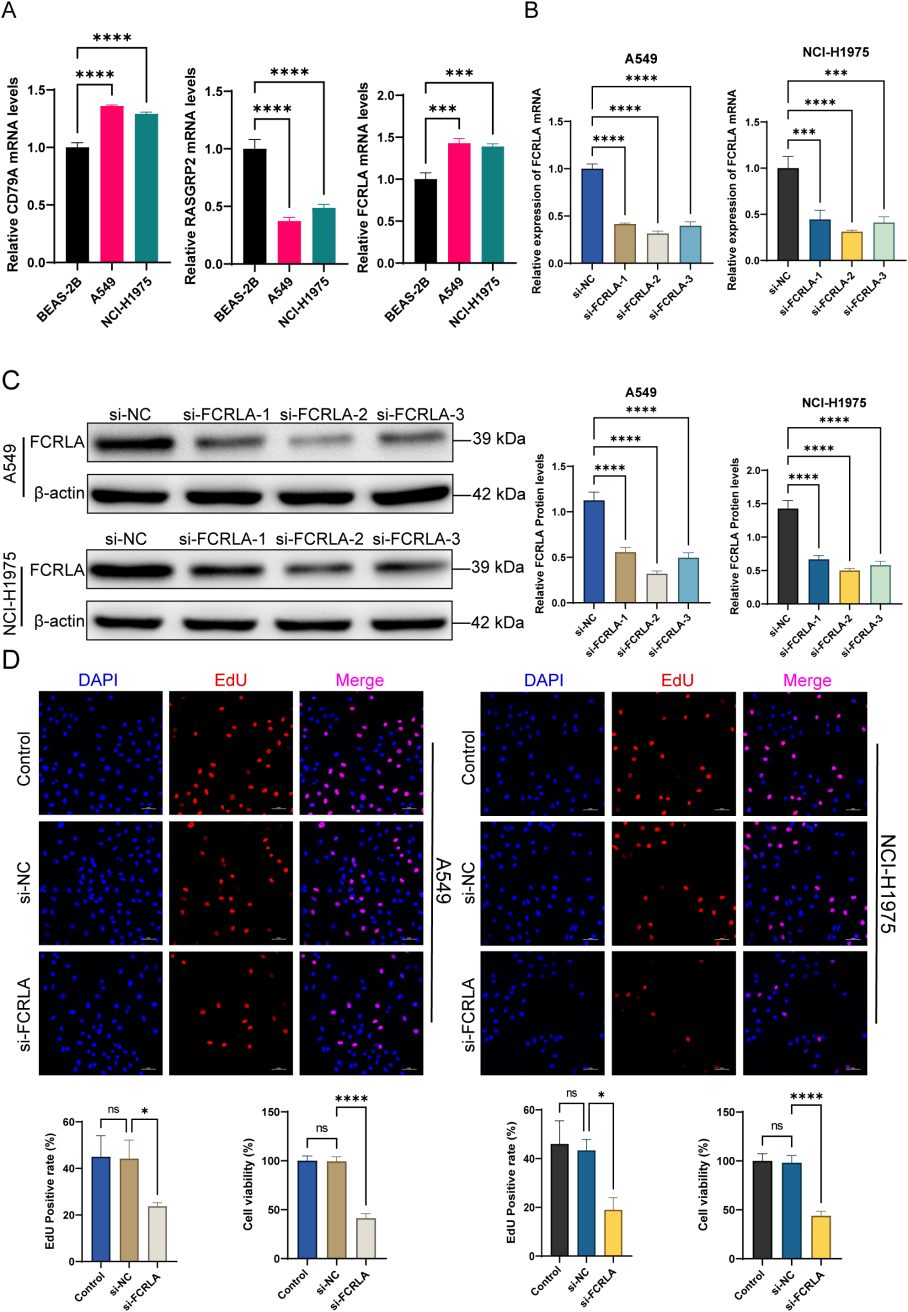
*FCRLA* knockdown inhibits the proliferation ability of LUAD cells. **(A)** The relative expression levels of *FCRLA*, *CD79A*, and *RASGRP2* mRNA in BEAS, A549, and NCI-H1975 cells were detected using RT-qPCR. **(B)** Changes in the relative expression levels of *FCRLA* mRNA 48 h after transfection with siRNA-1, 2, 3 interference sequences in A549 and NCI-H1975 cells were detected using RT-qPCR. **(C)** Relative FCRLA protein expression levels in A549 and NCI-H1975 cells 48 h after transfection with siRNA-1, 2, 3 interference sequences were detected using western blotting. **(D)** Changes in the proliferation ability of A549 and NCI-H1975 cells after different treatments were detected by EDU staining and CCK8 experiments (200×, scale bar: 100 μm). Compared between the two groups, *P < 0.05, ***P < 0.001, ****P < 0.0001, ns represents no statistical significance between the two groups.

Based on the association between the risk score and the tumor immune microenvironment revealed by the immune infiltration analysis, the role of mitochondrial dysfunction in LUAD cells and its relationship with *FCRLA* expression were further explored. As the energy center of cells, the functional state of the mitochondria is closely related to the immune response. Next, an in-depth analysis was conducted to examine the effect of mitochondrial dysfunction on the proliferation and necrosis of LUAD cells. To verify the accuracy of the bioinformatics prediction results, the changes in the expression level of *FCRLA* mRNA in human normal lung epithelial cells BEAS-2B and lung cancer cells A549 and NCI-H1975 were determined. *FCRLA* and *CD79A* were highly expressed in lung cancer cells, whereas *RASGRP2* was expressed at low levels. This finding was consistent with the bioinformatic results (**Figure 9A**). *FCRLA* expression in lung cancer cells was knocked down using siRNA to further verify the specific effects of FCRLA on lung cancer cells. All three siRNA sequences exhibited inhibitory effects on FCRLA mRNA and protein to varying degrees, indicating that a successful FCRLA knockdown cell line was constructed (**Figures 9B, C**). Si-FCRLA-2 knockdown was the most apparent. Therefore, in subsequent experiments, Si-FCRLA-2 was selected as the *FCRLA* knockdown sequence.

To further determine the effect of FCRLA on the phenotype of lung cancer cells, its effect on the proliferative ability of these cells was investigated using CCK8 and EDU staining. No significant difference was observed between the si-NC and NC groups, confirming that the transfection reagent did not affect cell proliferation. After FCRLA knockdown, the proportion of EDU decreased significantly, and cell proliferation was significantly inhibited. The CCK8 results also showed that cell viability decreased significantly after *FCRLA* knockdown. These results indicate that *FCRLA* knockdown inhibited the proliferation of lung cancer cells (**Figure 9D**).

### 3.9 *FCRLA* knockdown inhibits necroptosis in LUAD cells

**Figure 10:**
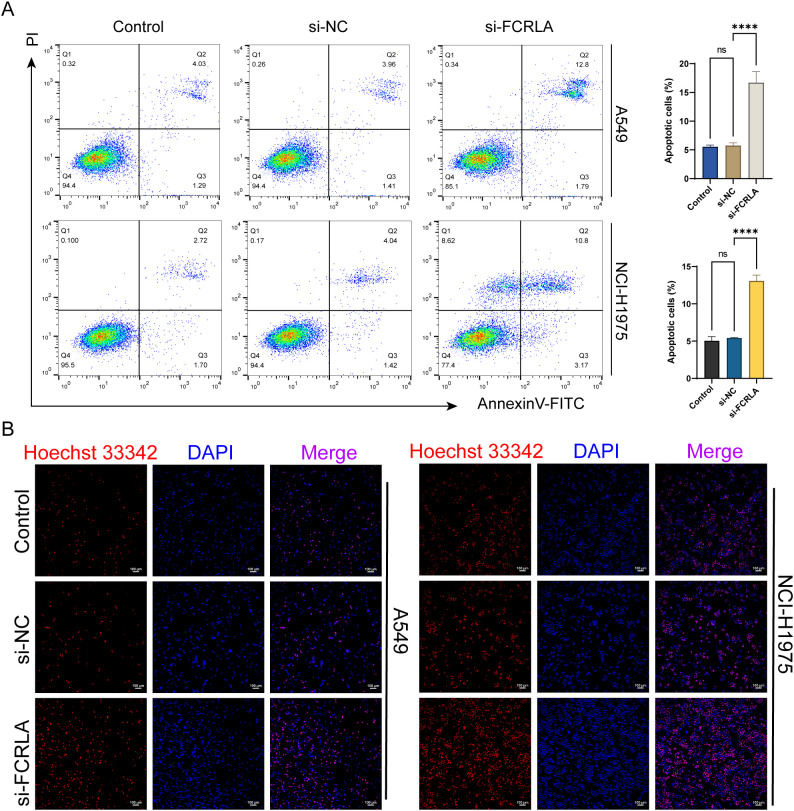
*FCRLA* knockdown induces necrosis in LUAD cells. **(A)** Changes in cell necrosis after treatment with each group of LUAD cells were detected using flow cytometry with FITC/PI double staining. **(B)** Changes in cell necrosis after treatment of each group in LUAD cells were detected using PI/Hoechst33342 cell viability staining (100×, Scale bar: 50 μm). Compared between the two groups, ****P < 0.0001, ns represents no statistical significance between the two groups.

**Figure 11:**
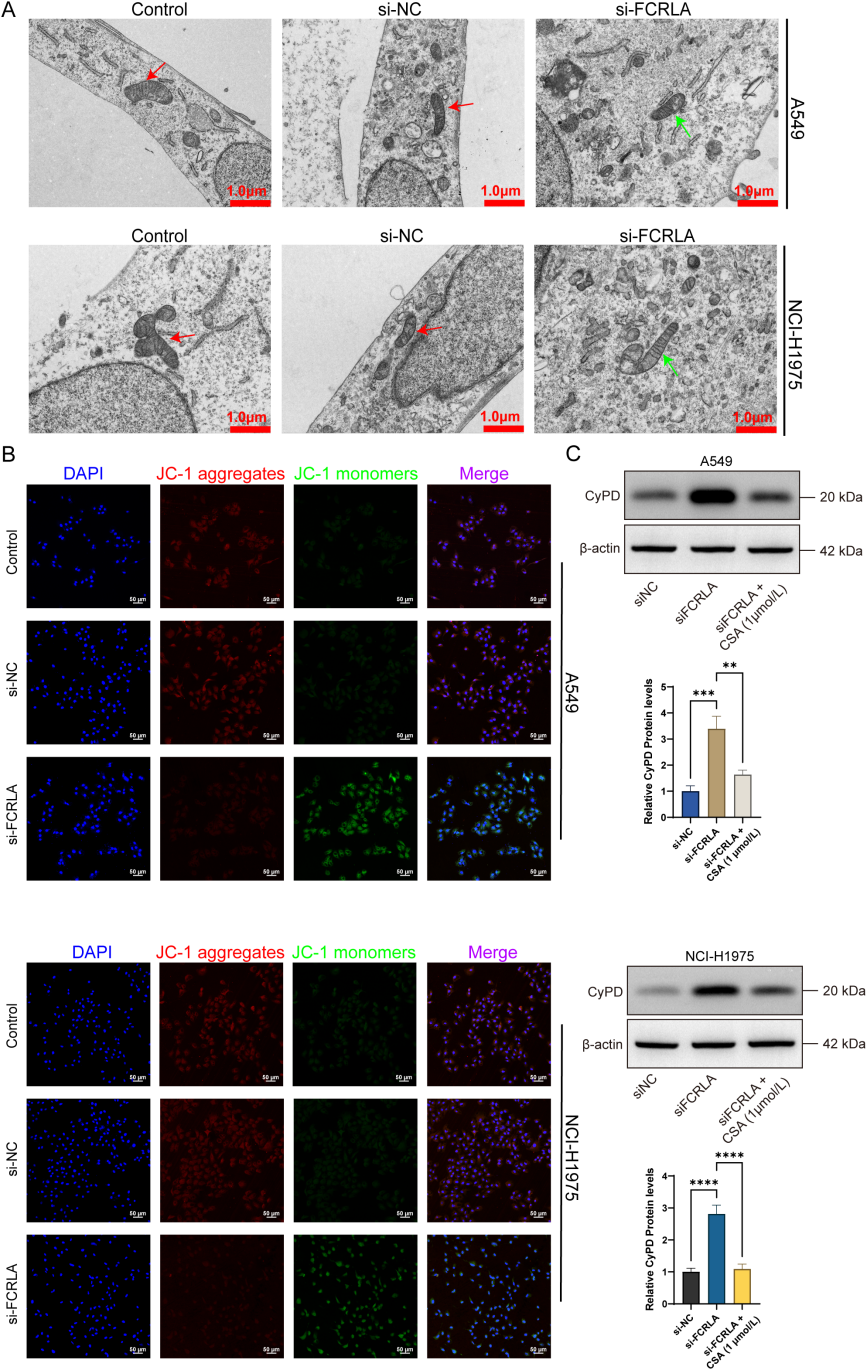
*FCRLA* knockdown leads to mitochondrial damage in lung cancer cells. **(A)** The changes in mitochondrial structure and morphology after treatment of each group in LUAD cells were observed using electron microscopy (10000×, scale bar: 1 μm). **(B)** The changes in mitochondrial membrane potential in A549 and NCI-H1975 lung cancer cells were detected using the JC-1 fluorescent probe (200×, Scale bar: 50 μm). **(C)** After *FCRLA* knockdown in A549 and NCI-H1975 cells, the cells were treated with cyclosporin A for 24 h, and the relative expression level changes of CypD protein were detected using western blotting. Compared between the two groups, **P < 0.001, ***P < 0.001, ****P < 0.0001.

To further confirm FCRLA as a mitochondria-driven necroptosis-related gene, the relationship between FCRLA and necroptosis was established in LUAD cells by performing necroptosis-related phenotypic experiments and observing changes in mitochondrial structure. In the detection of necroptosis, the si-FCRLA group showed significant differences compared to the si-NC group. Flow cytometry and FITC/PI double staining indicated that the proportion of necroptosis in the si-FCRLA group was significantly higher than that in the si-NC group (**Figure 10A**). Hoechst 33342/DAPI double staining indicated that cells in the si-FCRLA group showed apparent necroptosis characteristics, such as nuclear chromatin condensation and fragmentation, whereas such characteristics were less pronounced in the si-NC group (**Figure 10B**). Electron microscopy revealed that the mitochondrial structure of cells in the si-FCRLA group showed damage, such as mitochondrial swelling and cristae disorder, whereas the mitochondrial structure of the si-NC group was relatively intact (**Figure 11A**). In addition, JC-1 detection results showed that the mitochondrial membrane potential in the si-FCRLA group was significantly lower than that in the si-NC group, indicating mitochondrial dysfunction and further confirming the promotion of necroptosis. The siNC group showed no significant difference compared to the siNC group in each test (**Figure 11B**). *FCRLA* knockdown significantly enhanced the protein expression of CypD, and this effect was reversed by the addition of cyclosporin A (**Figure 11C**). These results suggest that FCRLA can protect against necrosis in LUAD cells, and this process may be closely linked to mitochondrial function.

## 4 Discussion

MPT-driven necrosis is defined as CypD-mediated programmed cell death, specifically necrosis. MPT-driven necrosis may lead to heart and neurological diseases, such as cerebral ischemia (29), and is associated with the prognosis of various tumors, including prostate cancer (30) and leukemia (31). However, the relationship between the MPTDNR lncRNAs and LUAD remains unclear. This study identified DEGs between 3,231 LUAD and adjacent samples. Taking the MPTDNRGs score as the phenotypic trait, the MPT-driven necrosis-related genes identified in the WGCNA analysis were intersected with the DEGs, resulting in 82 candidate genes. Using univariate Cox regression analysis and least absolute shrinkage and selection operator regression analysis, three prognostic genes were determined. Additionally, *in vitro* downregulation of FCRLA in LUAD cells induces necrosis.

The three prognostic genes identified in this study were *RASGRP2*, *CD79A*, and *FCRLA*. *RASGRP2*, a small guanosine triphosphatase of the RAS family (GTPases, including RAS and Rap), is a pivotal regulator of cell signaling.

*RASGRP2* was expressed at low levels in LUAD and was associated with the prognosis of patients with LUAD. *In vitro* cytological experiments demonstrated that *RASGRP2* inhibited the proliferation of LUAD cells by regulating mitochondria-dependent apoptosis (32), consistent with the present findings. The primary trigger factor for *RASGRP2* activation is calcium (33), and changes in mitochondrial permeability are associated with alterations in calcium ion concentrations. Mitochondrial permeability changes result from the accumulation of Ca^2+^ ions in the mitochondrial matrix, leading to the opening of the MPT pore, rendering it highly permeable to solutes and low-molecular-weight substances (34). This may be the mechanism by which RASGPR2 mediates MPT pore-driven cell necrosis. CD79A (CD79α) is a crucial component of the B-cell receptor complex, participating in the activation, proliferation, and differentiation of B cells in conjunction with CD79B (CD79β). In lung cancer, CD79A expression is primarily associated with regulating the tumor immune microenvironment. High CD79A expression may be related to the abundance of tumor-infiltrating B cells, suggesting its essential role in tumor immune surveillance and immune responses (35).

FCRL molecules belong to a large receptor family, homologous to the receptors of the immunoglobulin Fc portion. *FCRLA* is a prognostic gene in malignant tumors, such as ovarian (36), breast (37), and colorectal cancer (38). In this study, Spearman’s correlation analysis was conducted between prognostic genes and 11 immune checkpoints. The correlation between *FCRLA* and the immune checkpoint BTLA was the highest. Analysis of the correlation between the risk score and immune-infiltrating cells revealed a negative correlation with 15 immune cells and 12 immune cell functions. The immune cell and function scores of the low-risk group were lower than those of the high-risk group, indicating that the antitumor immune response levels in the high-risk group were lower than those in the low-risk group. Analysis of the gene expression levels of 11 conventional immune checkpoints revealed a negative correlation between the high- and low-risk groups. The expression levels of immune checkpoint genes in the high-risk group were low, with the strongest correlation observed between *FCRLA* and the immune checkpoint BTLA. Additionally, the immune, stromal, and microenvironment scores confirmed that the risk score had the strongest correlation with the immune score.

GSEA revealed that the top five pathways in the low-risk group were cytokine–cytokine receptor interaction, intestinal immune network for IgA production, hematopoietic cell lineage pathways, and chemokine signaling pathway. These signaling pathways are closely associated with processes such as tumor invasion, migration, and inflammatory immune responses. Growth factors and reactive cytokines produced during chronic inflammatory processes may lead to carcinogenesis (39). Cytokines exert their biological effects by binding to their corresponding receptors on the cell surface. After binding to their receptors, cytokines initiate complex intermolecular interactions within cells, ultimately leading to changes in gene transcription. The tumor necrosis factor receptor superfamily is a superfamily of cytokine receptors. Targeting regulatory T cells expressing the TNFR2 receptor is a safe and effective method for stimulating antitumor immunotherapy (40). The seven helical surface molecules bound by chemokines belong to two families, conventional and atypical chemokine receptors. The most extensively studied function of chemokine networks is cell migration, particularly leukocyte migration. Notably, cancer cells derived from non-leukocytes can evolve to express conventional chemokine receptors and respond to chemokines, promoting local invasion, spreading to draining lymph nodes, and metastasizing to distant tissues (41). *In vitro* experiments confirmed that downregulating *FCRLA* expression significantly inhibited the proliferation of LUAD cells, induced necroptosis in LUAD cells, and disrupted the stability of the mitochondrial membrane potential, resulting in damage to mitochondrial structure and morphology. Therefore, FCRLA may induce necrosis in LUAD cells through mitochondrial-driven pathways.

The innovation of this study lies in integrating the TCGA and Gene Expression Omnibus databases to identify MPTDNRGs associated with the prognosis of LUAD. Specifically, a prognostic risk model was constructed based on three key genes (*RASGRP2*, *CD79A*, and *FCRLA*), and *FCRLA* knockdown induced necrosis in LUAD cells by impairing mitochondrial function. This finding not only provides novel insights into the molecular mechanism of LUAD progression but also proposes potential therapeutic strategies targeting the MPT-driven pathway. Additionally, the correlation between *FCRLA* and immune checkpoints was investigated, revealing its potential role in regulating the tumor immune microenvironment. However, this study has some limitations. First, experimental validation was limited to *in vitro* cell models, and the *in vivo* effects of *FCRLA* knockdown on tumor growth and necrosis remain unclear. Second, the specific molecular mechanism by which *FCRLA* regulates MPT and necrosis in LUAD cells requires further investigation. Finally, the clinical relevance of the prognostic risk model should be verified in larger cohorts and ethnic populations. Future studies should focus on addressing these limitations through *in vivo* experiments, clarifying the detailed molecular pathways involved, and validating the applicability of this model in clinical settings.

In conclusion, this study identified FCRLA as a key regulator of MPTDN in patients with LUAD. FCRLA knockdown inhibits LUAD cell proliferation and induces necrosis by impairing mitochondrial function. These findings, supported by bioinformatic analysis and *in vitro* experiments, highlight the potential of FCRLA as a prognostic biomarker and therapeutic target. Future studies should validate these results *in vivo* and explore the detailed mechanisms of action of FCRLA in LUAD progression.

## Data Availability

The original contributions presented in the study are included in the article/**Supplementary Material**. Further inquiries can be directed to the corresponding author/s.

## Supplementary material

Supplementary Figure 1 Validation of the prognostic risk model in the GSE13213 dataset. **(A)** Risk curve showing the distribution of risk scores from low to high in the GSE13213 dataset. **(B)** Kaplan–Meier (KM) survival curve comparing the survival differences between high- and low-risk groups. **(C)** Heatmap displaying the expression levels of prognostic genes (*RASGRP2*, *CD79A*, and *FCRLA*) in high- and low-risk groups. **(D)** Receiver operating characteristic (ROC) curves for 1, 3, and 5 years, demonstrating the predictive accuracy of the risk model.
