# Supplementary figures and images for: Targeting FCRLA to induce necrosis in lung adenocarcinoma: a novel strategy for prognosis and therapy via MPT-Driven pathways
Source: Front Immunol. 2025 Aug 13;16:1596179. doi: 10.3389/fimmu.2025.1596179 (PMC12380818; doi:10.3389/fimmu.2025.1596179)

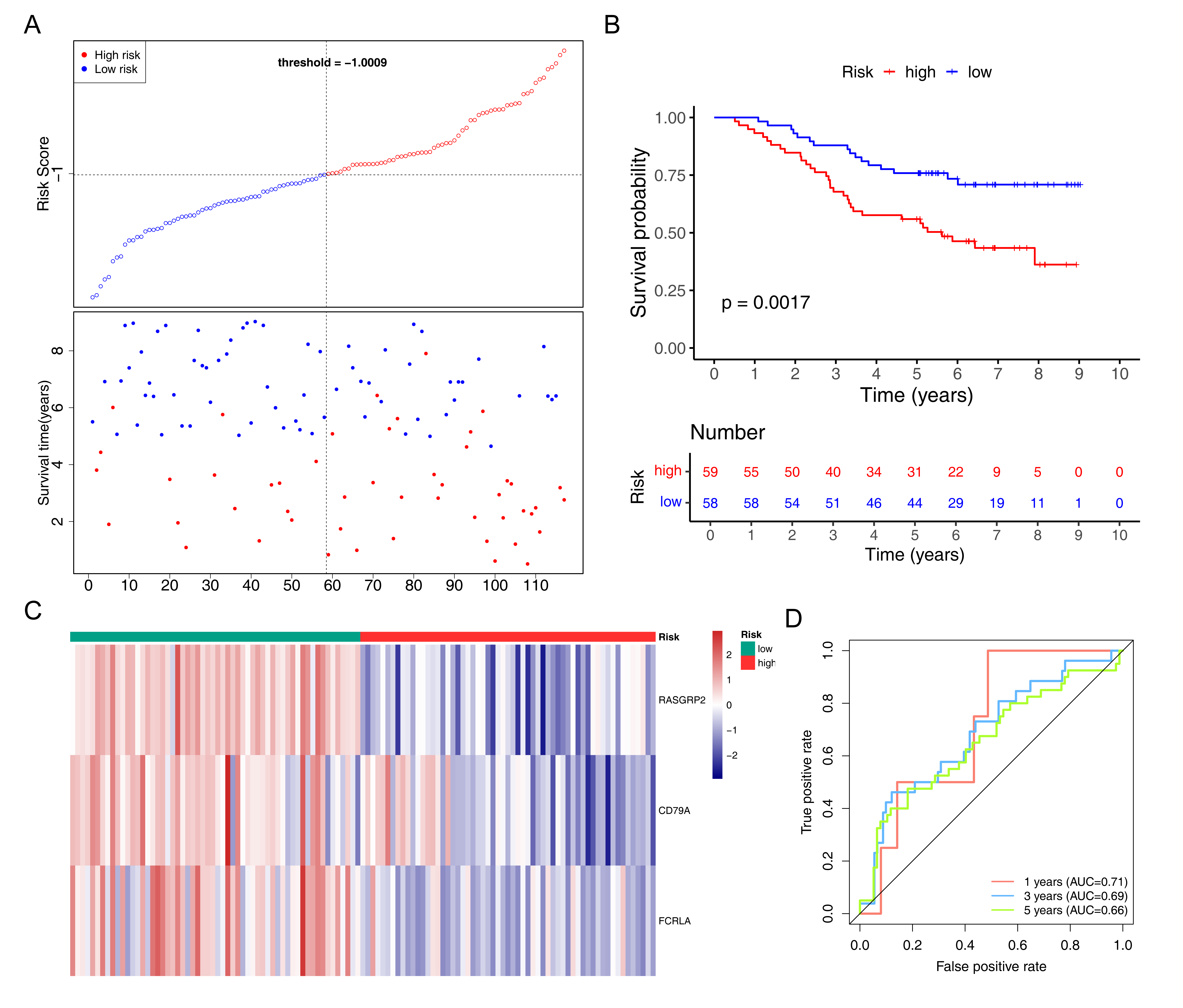

Supplement: Supplementary Figure 1 — Validation of the prognostic risk model in the GSE13213 dataset. (A) Risk curve showing the distribution of risk scores from low to high in the GSE13213 dataset. (B) Kaplan–Meier (KM) survival curve comparing the survival differences between high- and low-risk groups. (C) Heatmap displaying the expression levels of prognostic genes (RASGRP2, CD79A, and FCRLA) in high- and low-risk groups. (D) Receiver operating characteristic (ROC) curves for 1, 3, and 5 years, demonstrating the predictive accuracy of the risk model. [file Image1.tif]
